# Supplementary material for: Modeling Epithelial Homeostasis and Perturbation in Three-Dimensional Human Esophageal Organoids
Source: Biomolecules. 2024 Sep 5;14(9):1126. doi: 10.3390/biom14091126 (PMC11430971; doi:10.3390/biom14091126)
Supplement: Supplementary file 1 [file biomolecules-14-01126-s001.zip › biomolecules-3024430-Original Blot.pdf]

Supplementary Figure S4 B.
Original Blot

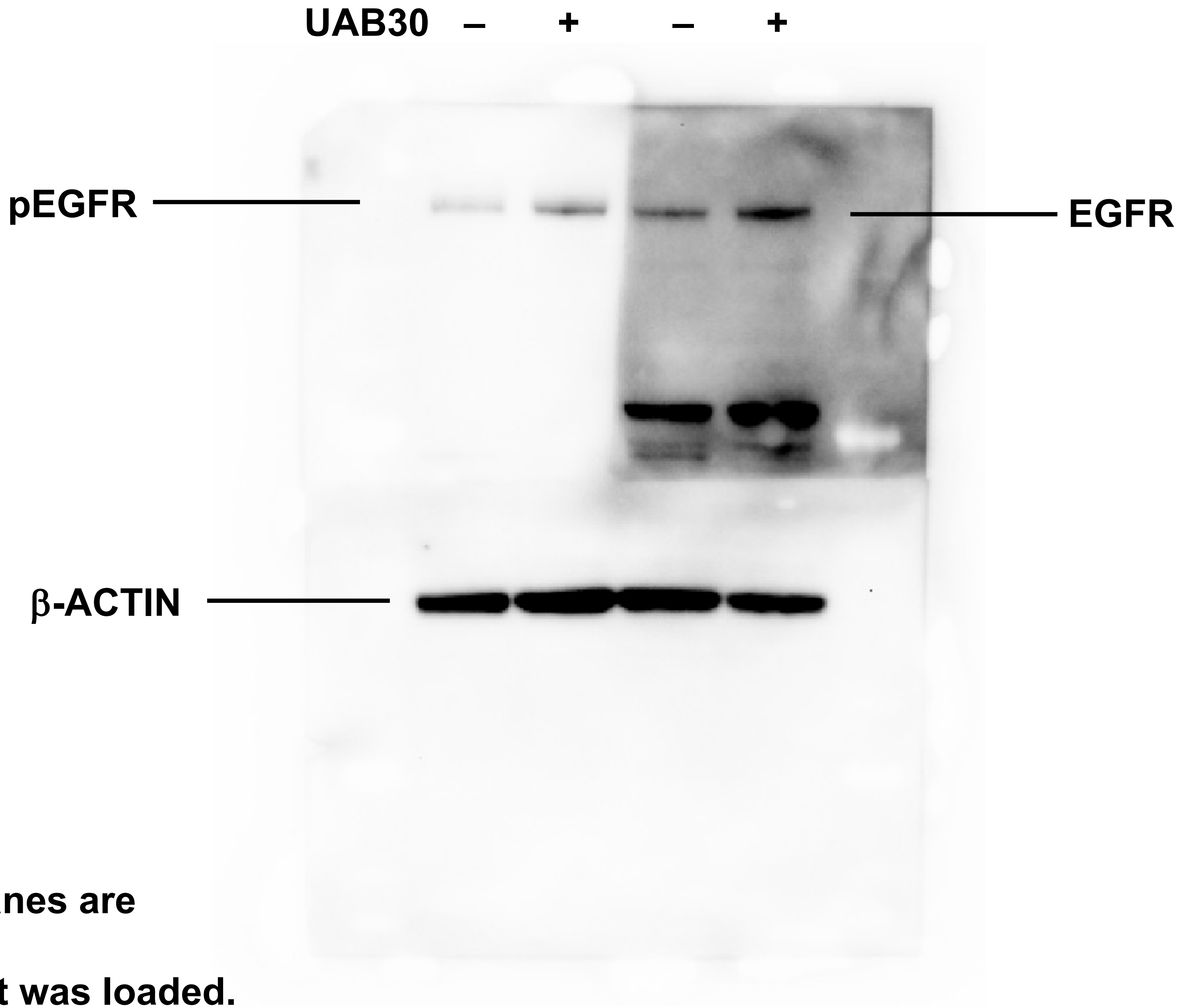

Left 2 lanes and right 2 lanes are technical replicate.
The same protein amount was loaded.
