# Supplementary material for: Modeling Epithelial Homeostasis and Perturbation in Three-Dimensional Human Esophageal Organoids
Source: Biomolecules. 2024 Sep 5;14(9):1126. doi: 10.3390/biom14091126 (PMC11430971; doi:10.3390/biom14091126)
Supplement: Supplementary file 1 [file biomolecules-14-01126-s001.zip › biomolecules-3024430-supplementary.pdf]

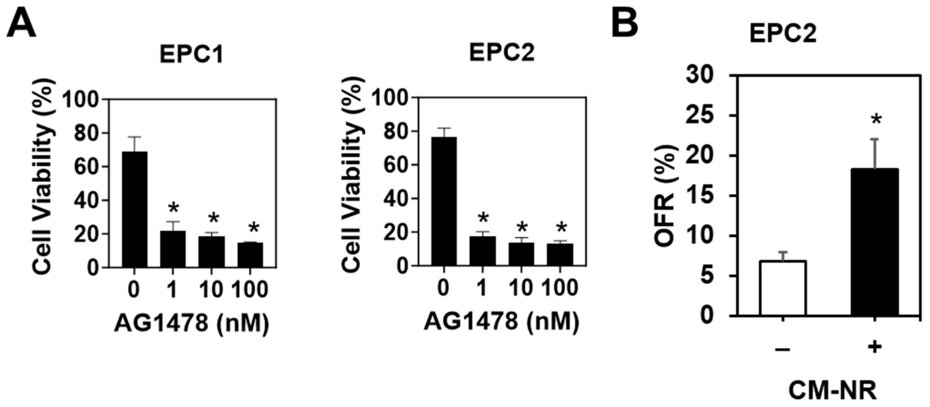


**Figure S1.** EGFR inhibitor AG1478 decreased the cell viability of organoids while CM-NR stimulate organoid formation. EPC1 and EPC2 organoids were grown using HOME0 in the presence or absence of AG1478 or CM-NR for 11 days and analyzed for cell viability in (A, One-way ANOVA) and OFR in (B, Student's t-test). * Indicates $p<0.05$.

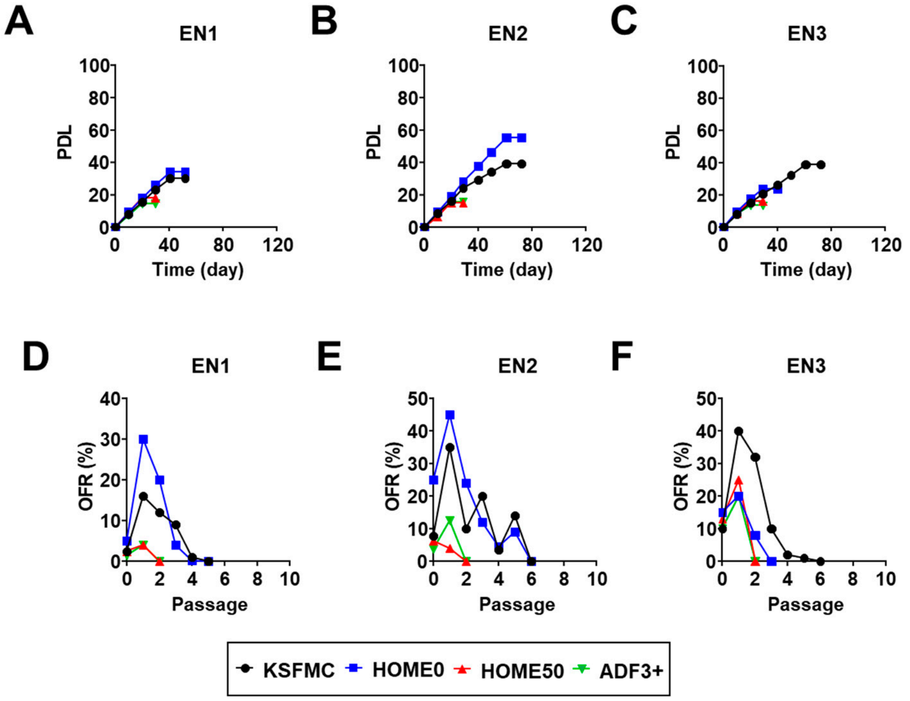


**Figure S2.** Normal esophageal PDOs display limited replicative lifespan. Endoscopic biopsies taken from three patients with normal esophageal mucosa (EN1-3) were dissociated and subjected to organoid culture with indicated media. The resulting organoids were passaged until they cease proliferation to determine PDL (**A-C**) and OFR (**D-F**) as plotted in representative graphs. Note that PDL was increased in KSFMC and HOME0 compared to HOME50 or ADF3+.

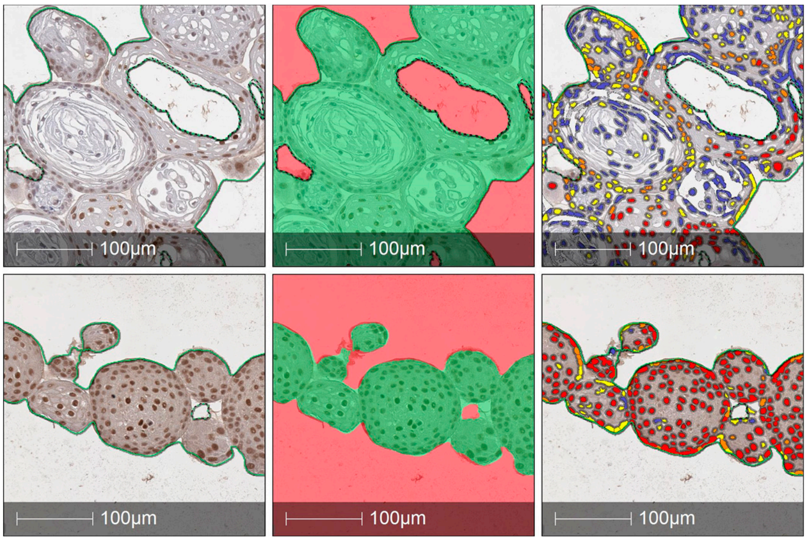


**Figure S3.** The representative image of using AI for image segmentation of individual organoids (indicated by green color in the images versus red background at second image panels) followed by identification of the intensity of each marker at individual cell levels. Red indicates cells with a high optical density of SOX2, red indicates cells with high optical density of SOX2, yellow indicates cells with the medium optical density of SOX2, blue indicates cells with low optical density of SOX2.

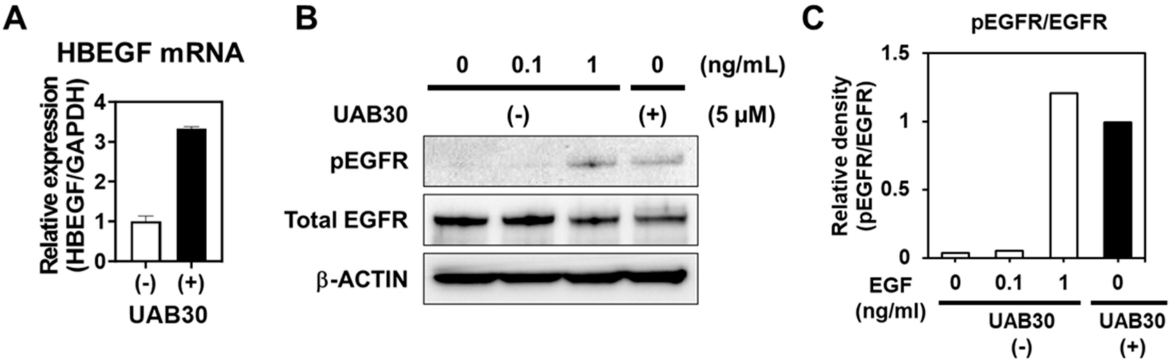


**Figure S4. UAB30 stimulated organoids display EGFR activation and HB-EGF mRNA induction.** EN1 PDO was grown in the HOME medium containing the indicated concentration of EGF concurrent with or without 5μM UAB30 for 11 days and subjected to quantitative RT-PCR assays to determine HBEGF mRNA (A) and immunoblotting for phospho-EGFR (Tyrosine 1068) and total EGFR (B) with densitometry as plotted in (C). GAPDH served as an internal control in (A). β-actin served as a loading control (B).
